# Early vs. Late Endovascular Extension Following Frozen Elephant Trunk Procedure: Effects on Clinical Outcomes and Aortic Remodeling

**DOI:** 10.3390/jcdd12030099

**Published:** 2025-03-14

**Authors:** Martin Wenkel, Nancy Halloum, Achim Neufang, Marco Doemland, Philipp Pfeiffer, Ahmad Ghazy, Chris Probst, Daniel-Sebastian Dohle, Hendrik Treede, Hazem El Beyrouti

**Affiliations:** Department of Cardiac and Vascular Surgery, University Medical Centre Mainz, Johannes Gutenberg University, 55131 Mainz, Germany

**Keywords:** thoracic aorta, chronic dissection, TEVAR, endovascular, FET

## Abstract

Background/Objectives: The frozen elephant trunk (FET) technique was introduced as a possible single-stage procedure for treating aortic arch pathologies. However, up to a third of patients are reported to need subsequent completion (extension). This retrospective analysis aimed to evaluate the impact of early (within 30 days; EC group) versus late (>30 days; LC group) endovascular completion with thoracic endovascular aortic repair (TEVAR) in patients treated with FET. Methods: A single-center, retrospective analysis of all consecutive patients for the period between June 2017 and December 2023 who underwent FET and received endovascular extension was conducted. Indications for endovascular extension were aneurysms of the descending aorta, aneurysmal progress, endoleak, malperfusion, distal stent-induced new entry (dSINE), and aortic rupture. Results: A total of 37 of 232 FET patients received endovascular extension (15.9%). Average age at the time of TEVAR was 63.3 ± 10.3 years. There was an increase in the maximum total aortic diameter post-FET from 40.8 ± 9 mm to 45.1 ± 14 mm prior to TEVAR. Only 14 patients (37.8%) had the desired complete occlusion of the false lumen or aneurysm prior to extension; 23 (62.2%) still had relevant perfusion of the false lumen or aneurysm. The EC and LC groups were defined by time between FET and TEVAR: a mean of 4.8 ± 5.2 days in the EC group and 18.4 ± 18 months in the LC group. The EC group had markedly more complex procedures, reflected in intensive care (10.7 ± 6.9 vs. 0.1 ± 0.3 days, *p* < 0.001) and hospitalization (22.4 ± 14.0 vs. 8.1 ± 5.6 days, *p* = 0.003) durations. There was one early death due to multiorgan failure in the EC group and there were none in the LC group. There were no major cardiac events in either group. In the EC group, seven patients (50%) suffered from postoperative respiratory failure and four (28.6%) developed acute kidney failure requiring dialysis. Only one patient in the LC group (4.3%) experienced complications. During follow-up, another three patients (21.4%) of the EC group died, but none of the LC group did. Post-extension aortic remodeling was similar in both groups, with complete occlusion achieved in 27 cases (72%) during early follow-up and increased to 90.6% after a mean of 22.0 ± 23.4 months. Conclusions: Following aortic arch repair using FET, there is still a need for second-stage repair in 16% of patients. Endovascular completion post-FET is safe and feasible with a technical success rate of 100%, but early completion is associated with greater morbidity and mortality. TEVAR extension surgery may be better delayed, if possible, until after recovery from the hybrid arch repair.

## 1. Introduction

Pathologies of the aortic arch and the descending aorta are a complex challenge for any surgeon [1,2,3]. For years, the elephant trunk technique was the method of choice in a multistage approach, followed by an additional open replacement of the thoracic or thoracoabdominal aorta [1]. With the development of stent-graft protheses, the frozen elephant trunk (FET) was introduced as a possible single-step procedure for treating aortic arch diseases without the need for further interventions [4,5,6,7]. However, up to a third of the patients treated with FET still need additional surgery to achieve sufficient aortic remodeling [8,9,10]. Open surgical repair of the downstream aorta following FET was previously the standard procedure [11]. However, with the advent of thoracic endovascular aortic repair (TEVAR), which has been shown to result in reduced mortality and morbidity, this approach has become increasingly obsolete [12,13]. This retrospective analysis aimed to evaluate the impact of an early versus a late endovascular completion with TEVAR in patients treated with FET.

## 2. Materials and Methods

This is a single-center, retrospective analysis. Patient data were collected from our own database. For the interval between both procedures, we differentiated between early reinterventions within the first 30 days after FET and late reinterventions with an interval longer than 30 days. For follow-up, we analyzed overall mortality, major cardiac adverse events (MACEs), stroke, respiratory and/or kidney failure, spinal cord injury, bleeding with the need of transfusion, and other vascular access-related complications. Additionally, we evaluated aortic remodeling, as seen in the latest available postoperative computed tomography angiograms (CTAs).

The study was conducted in accordance with the Declaration of Helsinki and ethical standards set forth by the institutional ethical committee. A waiver of informed consent was granted due to the retrospective and observational nature of the study (protocol code 25-00139, January 2025).

For all statistical analysis, we used IBM^®^ SPSS Statistics Software, Version 27.0.1 (Armonk, New York, NY, USA). Descriptive statistics were applied to describe the patients’ characteristics and continuous data were documented as medians (interquartile ranges) or means (±standard deviations). Categorical variables were described as numbers (percentages). We used the *t*-test for independent variables: *p*-values < 0.05 were considered statistically significant.

### 2.1. Patients and Follow-Up

All patients who received aortic surgery using the FET technique and who needed additional endovascular repair in our clinic were included. A total of 234 patients underwent aortic surgery using the FET technique in our clinic between June 2017 and December 2023, (169 male, mean 62.0 ± 10.9). Of those, two patients were excluded from this study: one because of subsequent open thoracoabdominal repair; a second because the endovascular extension was performed in another hospital. Until January 2024, a total of 37 of the remaining 232 patients received additional endovascular extension using TEVAR (15.9%). Patients were followed up in a routine manner.

### 2.2. Imaging and Surgical Procedure

Diagnosis was confirmed by multislice high-resolution spiral computed tomography angiography (CTA). Multiplanar and three-dimensional workstation reconstructions were used to evaluate aortic pathology. All patients had CTA before surgery, within the first few days after surgery, and were followed up routinely after 3, 6 and 12 months and at least yearly thereafter in our outpatient clinic. Images were evaluated with Sectra Workstation IDS 7 (Sectra AB, Linkoeping, Sweden), with automatic generation of centerline of flow (CLF) or with 3mensio software, version 10.4 (3mensio Medical Imaging BV, Utrecht, The Netherlands). Evaluation of the CTA included the largest diameter of the descending aorta, remaining perfusion of the false lumen or aneurysm, endoleak, and the angulation of the distal stent end in relation to the aortic wall.

Indications for endovascular reintervention were aneurysms of the descending aorta exceeding 5.5 cm, aneurysm progress, endoleak, malperfusion, distal stent-induced new entry (dSINE), and aortic rupture, as diagnosed using CTA [5]. When possible, informed consent was obtained and after anesthesiologic risk evaluation, patients were cleared for surgery. The surgeries were conducted under local or general anesthesia using either an open vascular access or minimal-invasive arterial puncture with a vascular closing device, depending on surgeon assessment. Using all available methods for minimizing radiation dose and contrast, a preoperative selected stent-graft with an anticipated oversizing of approximately 10% was implanted. If necessary, tapered stent-grafts or additional devices were used to fit the aorta or cover the necessary length for sufficient repair. After surgery, the patients were transferred routinely to an intermediate care unit or an intensive care unit, if the latter was necessary. All patients received prophylactic intravenous antibiotics for 5 days, the postoperative CTA was performed between postoperative days 2 and 5 and, depending on the patient’s recovery, patients were usually discharged between postoperative days 3 and 5.

Successful aortic remodeling was defined as complete false lumen thrombosis for patients with aortic dissection and as complete aneurysm occlusion for patients with aortic aneurysms. Residual false lumen perfusion or aneurysm perfusion were considered as unsuccessful aortic remodeling.

## 3. Results

In total, 24 of the 37 included patients were men (64.9%) (Table 1). The average patient age at the time of the TEVAR procedure was 63.3 (±10.3). A total of 31 patients (83.8%) had a history of hypertension, 5 patients (13.5%) an aneurysm of the infrarenal aorta, and 4 patients (10.8%) had a known coronary artery disease. A total of 4 patients (10.8%) had undergone vascular or endovascular surgery in the past, none of which were related to the disease of the aortic arch or descending aorta. Furthermore, 2 patients (5.4%) had Marfan’s disease (both in the LC group), 1 patient (2.7%) had diabetes, and another had obstructive lung disease. A total of 4 patients (10.8%) were current smokers; 10 patients (27.0%) were former smokers. In addition, 24 patients (64.9%) received platelet inhibitors and 7 (18.9%) received anticoagulants.

There were no relevant differences concerning age or medical history between both groups apart from those in a higher risk category of the physical status classification in the EC group.

Indications for the initial FET procedure were acute aortic dissection (51.3%) and aneurysm of the thoracic aorta (48.7%). Most devices were implanted in zone 2 (62.2%), followed by zones 0 and 1 (21.6% and 13.5%, respectively). In the majority of procedures, the Evita Open Plus and its successor Evita Open Neo (Artivion, Kennesaw, GA, USA) were used (89.2%). Other devices used were the Thoraflex (Terumo Aortic, Renfrew, UK), in two cases, and the straight Evita Neo and trifurcate Evita Neo in one case each. There were no relevant differences concerning primary outcome and aortic remodeling after TEVAR regarding implantation zones of the FET device. Compared to the first imaging after the FET procedure, there was an overall increase in the maximum diameter of the aorta at the distal end of the FET device from 40.8 ± 9 mm to 45.1 ± 14 mm (Table 2). Patients in the early-completion (EC) group had a smaller aortic diameter than patients with a late completion (LC) (41.7 vs. 47.3 mm), but the difference was not significant. Only 14 patients (37.8%) showed successful aortic remodeling prior to extension; 23 (62.2%) still had relevant perfusion of the false lumen or aneurysm.

### 3.1. Surgical Data

The EC and LC groups were defined by time between FET and TEVAR as either below or exceeding 30 days: a mean of 4.8 ± 5.2 days in the 14 patients (37.8%) of the EC group and 18.4 ± 18 months in the 23 (62.2%) the LC group (Table 3) was found. In total, 12 extensions were emergency procedures (32.4%), 9 (24.3%) were urgent, and the remaining 16 (43.2%) were elective. The average duration of all TEVAR procedures was 117.9 ± 59.7 min, with an average radiation dose of 3596 ± 3261 cGy*cm^2^ and 116.7 ± 69.9 mL of contrast used. In 23 procedures (62.2%), one stent-graft was sufficient; 13 patients (35.1%) needed two devices; and three devices were necessary in one patient (2.7%). The stent-grafts used varied in diameter from 28 mm as the smallest to 42 mm as the largest and from 99 mm to 209 mm in length; eight stent-grafts (21.6%) were tapered grafts. Cut-down vascular access was used in 24 (64.9%) cases and percutaneous access in 13 (35.1%). Prophylactic cerebrospinal fluid (CSF) drainage to prevent spinal cord injury was used in four cases (10.8%). All interventions had technical success and there was no need for conversion to open surgery.

A total of 14 (37.9%) patients needed early endovascular completion (within 30 days of FET): 8 (57.1%) of these completions were emergency surgeries, 5 (35.7%) urgent, and 1 (7.1%) elective. A type Ib endoleak with relevant remaining distal entry flow was responsible for five extension procedures (35.7%). In four cases (28.6%), extension was necessary due to spinal or visceral malperfusion. Other indications were as follows: intended two-stage procedures (n = 3, 21.4%), aortic rupture, and dSINE in one case each (7.1%). The early-completion procedure was typically more demanding: duration 146 vs. 108 min; radiation dose 4104 vs. 3286 cGy*cm^2^; contrast used, 132 vs. 101 mL.

**Figure 1:**
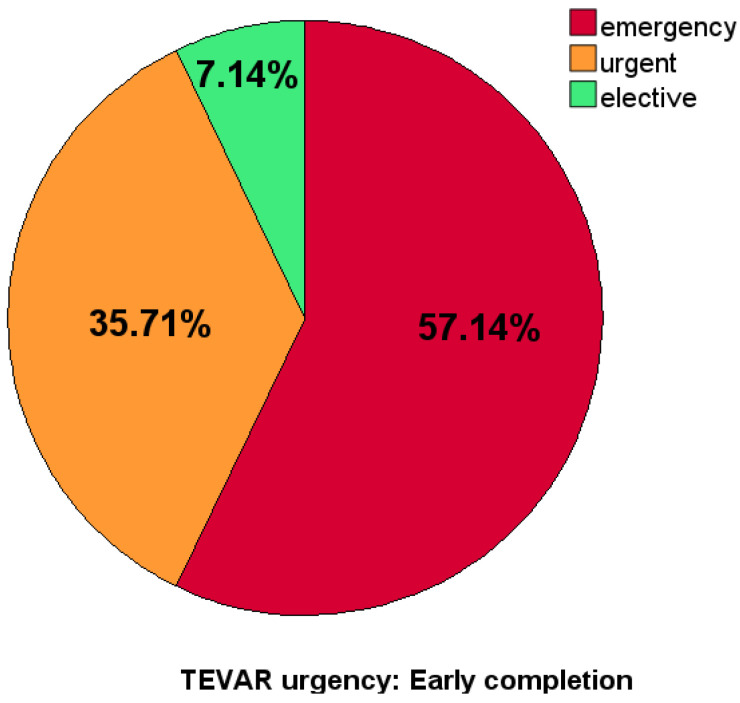

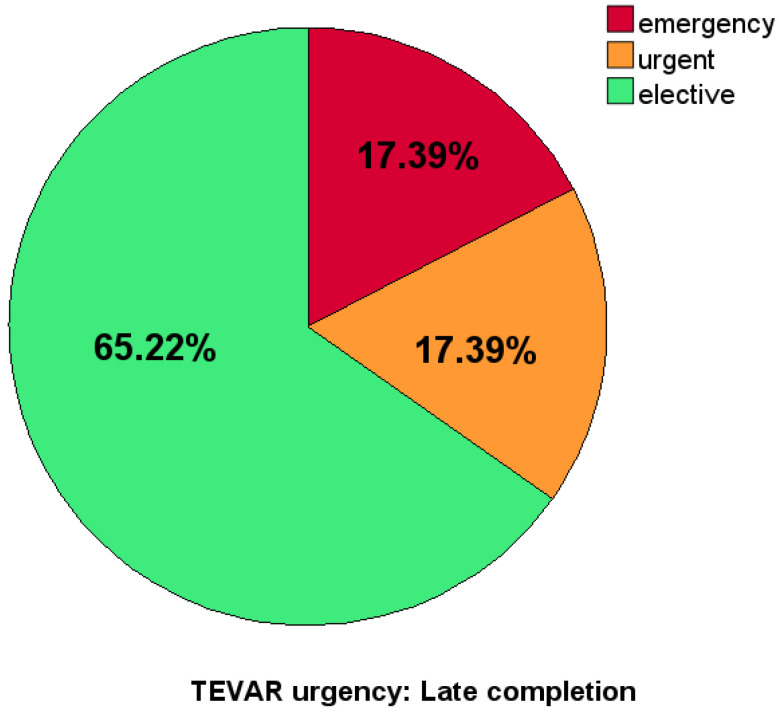
Acuity of TEVAR extension procedure post-FET in early- and late-completion groups.

In comparison, the LC group each comprised 4 (17.4%) emergency and urgent procedures and 15 (65.2%) elective ones. Relevant endoleak and dSINE (isolated or in combination) were indications for second-stage repair in 13 patients (56.5%); two-stage repair was planned for 3 patients (13.0%). Two patients (8.7%) suffered renovisceral malperfusion and there was one case with aortic rupture (Figure 1).

### 3.2. Early Follow-Up and Outcomes

Overall, patients stayed in intensive care for 3.9 ± 6.5 days and in hospital for an average of 13.5 ± 11.9 days. Again, the EC group had significantly longer values, reflecting more challenging procedures: 10.7 days intensive care vs. 0.1 days; 22.4 days hospitalization vs. 8.1 days.

During the first 30 days, there was one death due to multiorgan failure in the EC group (none in the LC group). There were no major cardiac events in either group. However, in the EC group, seven patients suffered from postoperative respiratory failure (50%) and four developed acute kidney failure requiring dialysis (28.6%), both of which were significant from the LC group. There was one bleeding complication in each group: the single LC bleeding complication was due to a pseudoaneurysm on the access vessel. In summary, a single LC patient (4.3%) experienced early complications.

Postoperative CTAs showed endoleak in both groups, but none of which were in need of reintervention during that stay and were treated conservatively (Table 4).

Complete occlusion of the false lumen or aneurysm was achieved in 27 cases (72%). There was a higher, but not significant, rate of complete occlusion in the EC group (92.3%) compared to group B (65.2%).

### 3.3. Mid-Term Follow-Up

**Figure 2:**
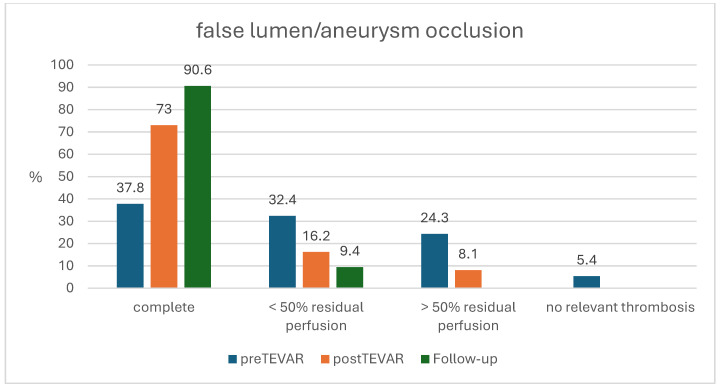
Aortic remodeling post-TEVAR extension.

Follow-up beyond 30 days was available for 33 of the 37 patients and was a mean of 22.0 ± 22.9 months and was longer in the EC group, with a mean of 24.7 vs. 20.5 months. During mid-term follow-up, another three EC patients died (due to sepsis or respiratory failure) but no LC patient did. However, one LC patient suffered a transient ischemic attack 4 months after TEVAR. Another patient’s aorta, with only a partly occluded false lumen during the early follow-up, ruptured after 4 months and needed additional emergency TEVAR. CTAs showed type 1b endoleak in four patients as well as one type II endoleak in another patient and one patient with dSINE. This led to another three reinterventions (EC 1, LC 2). Complete occlusion of the false lumen or aneurysm was achieved in 29 cases (90.6% overall; 80% EC; 95.5% LC) (Table 5, Figure 2).

## 4. Discussion

If left untreated, aortic pathologies are linked to a high death rate; in particular, type A aortic dissections (TAADs) can have a 30-day mortality rate of up to 90% [14].

**Figure 3:**
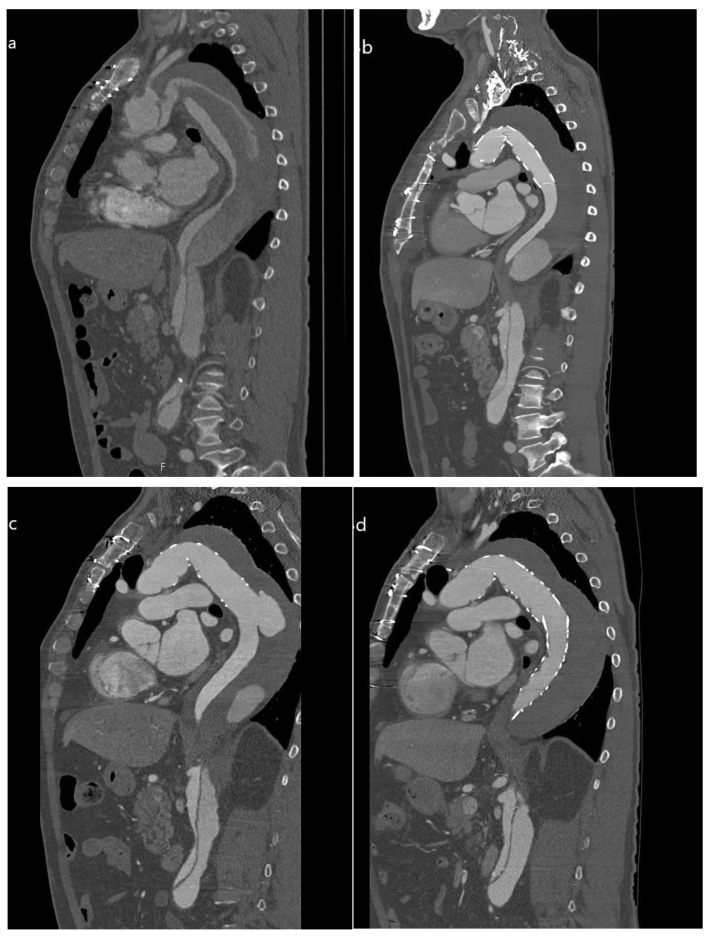
Computed tomography images of patient with acute type A aortic dissection treated with FET and subsequent TEVAR extension to address dSINE and aneurysmal growth. Patient with acute type A aortic dissection (**a**) was treated with FET. First postoperative imaging (**b**) showed satisfactory result, but follow-up imaging after 3 months (**c**) revealed dSINE with resulting aneurysmal growth requiring second-stage repair (**d**) with sufficient positive aortic remodeling.

The treatment of pathologies of the aortic arch, such as aneurysms or aortic dissections, is a challenge for any surgeon. For the complete treatment of the diseased aorta, elephant trunk surgery has been used—since it was first described by Borst et al. in 1983— to enable a multistage approach with the open repair of the ascending aorta and the aortic arch in a first stage and open thoracoabdominal repair in a second stage [1]. Due to the invasiveness of both surgeries, the combined procedures had both a high morbidity and mortality. With the development of stent-graft protheses in the early 90s, the frozen elephant trunk was described by Suto et al. in 1996 as a possible single-stage treatment without the need for additional open thoracoabdominal repair [4]. It showed a significant lower in-hospital mortality rate compared to the conventional elephant trunk, is now the state of the art in treating aortic diseases with the involvement of both the aortic arch and descending aorta, and is well established in the current guidelines [3,5,15,16,17,18]. However, it did not live up to all expectations, as there is still a need for additional surgery in up to a third of patients treated with FET [9]. The desired aortic remodeling is not achieved in every case as regards sufficiently preventing further aneurysm growth or aortic rupture. Further progress of the disease is usually detected during routine follow-up, but might manifest itself beforehand, or is even expected in advance. In those cases, the FET prothesis provides an ideal landing zone for TEVAR as a minimally invasive technique without the high morbidity or mortality of an open aortic repair, and the combination of both techniques is well established (Figure 3) [8,11,15,16,17]. However, there are few data evaluating the influence of the interval between procedures, and this single-center analysis goes some way to address that.

In our study, out of 232 patients treated with FET, 37 (15.9%) needed subsequent endovascular extension. This portion matches the reintervention rates reported by other authors, which range from 9 to 33% [3,8,9,10,16,18,19,20]. Technical success was achieved in every extension and there was no conversion to open surgery, highlighting the safety and feasibility of the procedure. However, we discovered important differences between patients treated within 30 days of FET and patients extended after a longer interval. When treated early, patients had higher mortality (28.6% vs. 0%) and significantly more complications such as respiratory failure (50% vs. 0%), acute kidney failure (28.6% vs. 0%), sepsis (14.3% vs. 0%), or spinal cord injury (7.1% vs. 0%). Patients were treated longer on ICU and stayed longer in hospital. Although not significant, the amount of emergency surgeries was higher, surgeries took longer, patients and staff were exposed to more radiation, and patients received more contrast agent than when treated after 30 days. However, it is not possible to distinguish the higher mortality and complication rates originating from the TEVAR extension or the index FET procedure: most likely, it is a combination of both and the added risks of an emergency procedure. The patients were still in recovery from major cardiovascular surgery, some still in an unstable condition, when another major complication such as aortic rupture or visceral malperfusion occurred. Thus, the correlation between an early endovascular repair and a higher mortality and morbidity is quite understandable.

Despite this, Fortin and al. reported 0% in-hospital mortality and 100% survival during 23 months of follow-up for a series of TEVARs 1 month post-FET [21]. In several published papers in the literature with follow-up varying from 18 to 58 months, survival for TEVAR later than one month post-FET varies from 87.5 to 100% and in-hospital mortality varies from 0 to 12.5% [22,23,24,25]. However, the literature reflects a high degree of variability, even in what procedures are described as FET, especially when using a dedicated FET prothesis or not, as well as regards which one is used. It should also be noted that there is as yet no standard for describing planned and unplanned FET completion procedures.

In this study, patients with late endovascular repair had wider aortic diameters than patients with early completion as the desired complete occlusion of the false lumen or aneurysm was not achieved as intended and the diseased vessel was still under the influence of the aortic blood pressure. Over an interval of 18.4 months, the aortic diameter at the distal end of the FET prothesis grew a mean 4 mm and the desired complete occlusion was only achieved in 37.8%, confirming that FET alone is not sufficient for aortic remodeling in every case.

**Figure 4:**
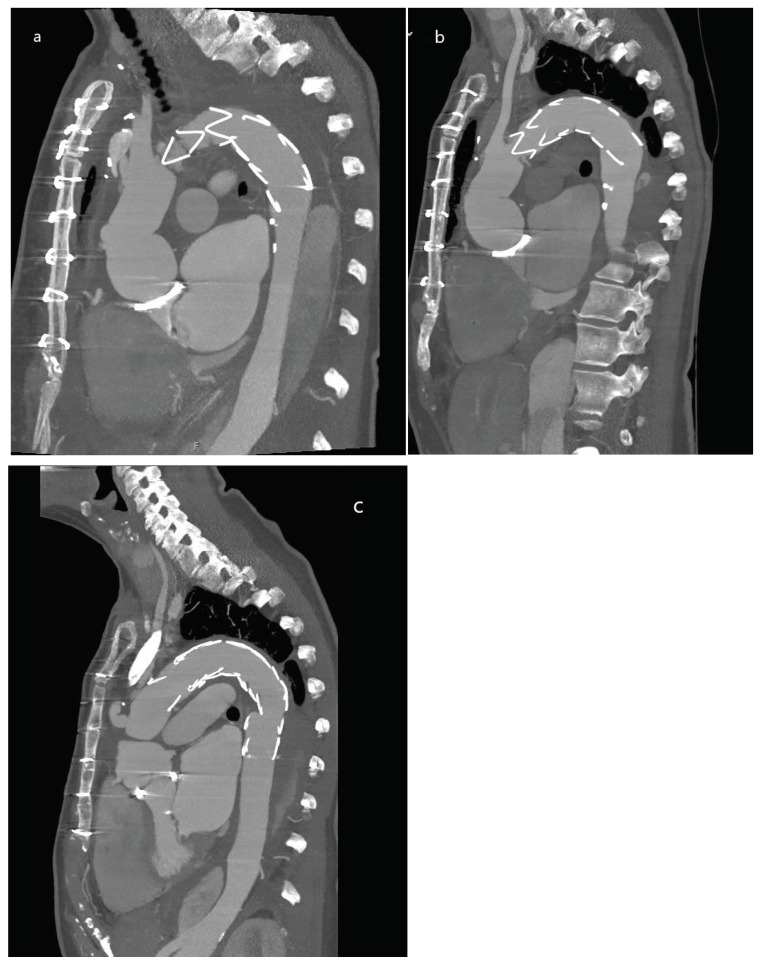
Computed tomography images of patient with aortic arch aneurysm treated with FET and subsequent TEVAR extension to address distal bird beak. Patient with aortic arch aneurysm treated with FET (**a**) who subsequently experienced increase in distal angle of FET prothesis, leading to a distal bird beak (**b**), which was addressed with TEVAR extension (**c**).

A possible explanation could be that, compared to the first postoperative CT angiograms, there was an increase of a mean 15° in the angulation between the distal stent end of the FET prothesis and the native aorta, forming a distal bird beak sign in 17 out of the 23 patients (73.9%, Figure 4). This facilitates the development of retrograde entry flow, and increased blood flow prevents the desired occlusion and remodeling. Furthermore, a steeper angulation may cause more friction and mechanical stress to an already weakened aortic wall and thus may cause dSINE or even aortic rupture. Whether this aspect is related to length, implantation zone, or the rigidity of the FET prothesis itself will be evaluated in another analysis. As indications for the FET surgery included acute aortic diseases such as TAADs as well as chronic aortic diseases with large aneurysms, the interpretation of aortic growth is difficult. Our cohort is not big enough to form significant subgroups.

Liebrich et al. have already described a higher rate of secondary aortic interventions when the distal anastomosis was performed in zone 2 and a short stent-graft was chosen [20]. However, a more distal landing zone with more aortic coverage is associated with a higher risk of spinal cord injury [26,27,28]. The increase in the aortic diameter and angulation after successful FET procedure during follow-up confirms and highlights the necessity for regular CTAs to detect patients at risk of aortic rupture. After completion with TEVAR, the rate of complete occlusion of the false lumen thrombosis or the aneurysm increased from 34.8% to 72.9% in an early follow-up and further increased to 90.6% after 22 months. Only in three patients (8.1%) was a third procedure necessary.

## 5. Conclusions

Following aortic arch repair using the frozen elephant trunk technique, there was still a need for second-stage repair in around 20% of the patients. Endovascular completion using TEVAR is safe and feasible, with a technical success rate of 100%. However, second-stage surgery may be better delayed, if possible, until after the patients’ recovery from the open arch repair as patients have a higher mortality and morbidity in the early stage.

## Figures and Tables

**Table 1:** Patient characteristics and comorbidities.

	All	Early Completion	Late Completion	*p*-Value
n	37	14 (37.8%)	23 (62.2%)	
Male	24 (64.9%)	11 (78.6%)	13 (56.5%)	
Age at FET (years)	62.3 ± 10.5	62.8 ± 12.1	62.1 ± 9.3	0.860
Age at TEVAR (years)	63.3 ± 10.3	62.8 ± 12.1	63.6 ± 9.0	0.834
BMI (kg/m^2^)	25.8 ± 4.6	27.2 ± 3.2	24.9 ± 5.0	0.135
Comorbidities				
Arterial hypertension	31 (83.3%)	10 (71.4%)	21 (91.3%)	0.118
Diabetes	1 (2.7%)	0	1 (4.3%)	0.443
CAD	4 (10.8%)	1 (7.1%)	3 (13.0%)	0.587
COPD	1 (2.7%)	1 (71%)	0	0.336
CKD	4 (10.8%)	2 (14.3%)	3 (13.0%)	0.629
Stroke	6 (16.2%)	3 (21.4%)	3 (13.0%)	0.539
Smoking history				
Former or current	14 (37.8%)	5 (35.7%)	9 (39.1%)	0.841
Atrial fibrillation	3 (8.1%)	1 (7.1%)	2 (8.7%)	0.871
Anticoagulants and platelet inhibitors				
DOAC or coumadin	7 (18.9%)	2 (14.3%)	5 (21.7%)	0.573
Platelet inhibitors	24 (64.9%)	3 (21.4%)	21 (91.3%)	<0.001
Abdominal aneurysm	5 (13.5%)	1 (7.1%)	4 (17.4%)	0.349
Previous vascular or endovascular surgery	4 (10.8%)	0	4 (17.4%)	0.104
ASA I	0	0	0	
ASA II	4 (10.8%)	2 (14.3%)	2 (8.7%)	0.607
ASA III	14 (37.8%)	0	14 (60.9%)	0.206
ASA IV	19 (51.4%)	12 (85.7%)	7 (30.4%)	0.145

Values are mean, standard deviation, or n (%). ASA, American Society of Anesthesiologists Physical Status Classification; BMI, body mass index; CAD, coronary artery disease; CKD, chronic kidney disease; COPD, chronic obstructive pulmonary disease; DOAC, direct oral anticoagulants; FET, frozen elephant trunk; TEVAR, thoracic endovascular aortic repair.

**Table 2:** Aortic diameters after index FET and before and after subsequent TEVAR extension.

	All	Early Completion	Late Completion	*p*-Value
Diameter post-FET (mm)				
Aneurysm diameter/Aorta (Total)	39.9 ± 9.7	38.4 ± 7.8	40.6 ± 10.3	
True lumen	20.7 ± 8.5	17.3 ± 7.2	23.3 ± 8.6	
False lumen	20.8 ± 11.5	21.1 ±10.4	20.5 ± 12.2	
Diameter pre-TEVAR (mm)				
Aneurysm diameter/aorta (total)	45.9 ± 14.1	46.2 ± 18.8	45.8 ± 11.0	
True lumen	24.7 ± 14.7	17.3 ± 7.2	30.1 ± 16.3	
False lumen	19.6 ± 14.7	21.1 ± 10.4	18.5 ± 17.1	
Diameter post-TEVAR (mm)				
Aneurysm diameter/aorta (total)	39.6 ± 10.9	31.6 ± 7.7	42.9 ± 10.3	
True lumen	23.7 ± 9.7	26.0 ± 12.4	22.1 ± 6.6	
False lumen	13.8 ± 10.9	12.1 ± 11.2	15.1 ± 10.6	
Diameter follow-up (mm)				
Aneurysm diameter/aorta (total)	39.7 ± 8.3	38.3 ± 9.9	40.2 ± 7.6	
True lumen	24.2 ± 4.9	24.5 ± 3.5	24.0 ± 5.5	
False lumen	16.4 ± 12.6	14.8 ± 11.7	17.2 ± 13.0	
Angulation post-FET	20.9° ± 33	20.0° ± 33	21.4° ± 33	0.909
Angulation pre-TEVAR	30.7° ± 35	20.0° ± 33	36.7° ± 35	0.183

Values are mean or standard deviation. FET, frozen elephant trunk; TEVAR, thoracic endovascular aortic repair.

**Table 3:** Extension procedures outcomes.

	All	Early Completion	Late Completion	*p*-Value
Interval between surgeries	11.4 ± 16.7 months	4.8 ± 5.2 days	18.4 ± 18.0 months	
Urgency				
Emergency	12 (32.4%)	8 (57.1%)	4 (17.4%)	0.277
Urgent	9 (24.3%)	5 (35.7%)	4 (17.4%)	0.757
Elective	16 (43.2%)	1 (7.1%)	15 (65.2%)	0.193
Indication				
Intended 2-stage	6 (16.2%)	3 (21.4%)	3 (13.0%)	
Endoleak	8 (21.6%)	3 (21.4%)	5 (21.7%)	
dSINE	6 (16.2%)	1 (7.1%)	5 (21.7%)	
Rupture	2 (5.4%)	1 (7.1%)	1 (4.3%)	
Aneurysm growth	1 (2.7%)	0	1 (4.3%)	
Malperfusion	6 (16.2%)	4 (28.6%)	2 (8.7%)	
Endoleak + dSINE	3 (8.1%)	0	3 (13.0%)	
Endoleak + aneurysm growth	3 (8.1%)	2 (14.3%)	1 (4.3%)	
Other	2 (5.4%)	0	2 (8.7%)	
Duration of surgery (min)	118 ± 60	146 ± 110	108 ± 110	0.068
Contrast used (mL)	117 ± 70	132 ± 100	101 ± 100	0.383
Radiation dose (cGy*cm^2^)	3596 ± 3261	4105 ± 2552	3286 ± 2552	0.506
TEVAR diameter (mm)	31 (24–44)	31 (28–42)	36 (24–44)	0.758
TEVAR length (mm)	164 (94–209)	164 (94–209)	164 (104–209)	0.576
Number of TEVAR devices used				
1 device	23 (62.2%)	11 (78.6%)	12 (52.2%)	0.114
2 devices	13 (35.1%)	3 (21.4%)	10 (43.5%)	0.183
3 devices	1 (2.7%)	0	1 (4.3%)	0.443
Tapered TEVAR	8 (21.6%)	2 (14.3%)	6 (26.1%)	0.412
Vascular access				
Cut-down	24 (64.9%)	11 (78.6%)	13 (56.5%)	0.183
Percutaneous	13 (35.1%)	3 (21.4%)	10 (43.5%)	0.004
CSF drainage	4 (10.8%)	1 (7.1%)	3 (13.0%)	0.294
Technical success	37 (100%)	14 (100%)	23 (100%)	
Conversion	0	0	0	
Concomitant procedure	6 (16.2%)	5 (35.7%)	1 (4.3%)	0.040

Values are mean, standard deviation (except TEVAR which are median, range), or n (%). dSINE, distal stent-induced new entry; TEVAR, thoracic endovascular aortic repair.

**Table 4:** Follow-up and outcomes.

	All	Early Completion	Late Completion	*p*-Value
Mortality				
30-day mortality (n = 37)	1 (2.7%)	1 (7.1%)	0	0.336
Late mortality (n = 33)	3 (9.1%)	3 (23.1%)	0	0.038
Intensive care (days)	3.9 ± 6.5	10.7 ± 6.9	0.1 ± 0.3	<0.001
Hospitalization (days)	13.5 ± 11.9	22.4 ± 14	8.1 ± 5.6	0.003
Follow-up (months)	22.0 ± 22.9	24.7 ± 24.6	20.5 ± 21.8	0.497
30-day complications				
Myocardial infarction	0	0	0	
Stroke	1 (2.7%)	1 (7.1%)	0	0.336
Bleeding	2 (5.4%)	1 (7.1%)	1 (4.3%)	0.741
Respiratory failure	7 (18.9%)	7 (50.0%)	0	0.003
Acute kidney failure	4 (10.8%)	4 (28.6%)	0	0.040
Spinal cord injury	1 (2.7%)	1 (7.1%)	0	0.336
Surgical site infection	0	0	0	
Sepsis	2 (5.4%)	2 (14.3%)	0	0.165
Pseudoaneurysm	1 (2.7%)	0	1 (4.3%)	0.328
Complications during mid-term follow-up (n = 33)				
Myocardial infarction	0	0	0	
Stroke	1 (3.1%)	0	1 (4.3%)	0.329
Aortic rupture	1 (3.1%)	0	1 (4.3%)	0.329
Graft migration	0	0	0	
Graft infection	0	0	0	
Reintervention	4 (12.5%)	1 (7.1%)	3 (13.0%)	0.805

Values are mean, standard deviation, or n (%).

**Table 5:** Aortic remodeling and false lumen/aneurysm occlusion.

	All	Early Completion	Late Completion	*p*-Value
Pre-TEVAR CTA	n = 37	n = 14	n = 23	
Complete occlusion	14 (37.8%)	6 (42.5%)	8 (34.8%)	0.635
<50% residual perfusion	12 (32.4%)	3 (21.4%)	9 (39.1%)	0.277
>50% residual perfusion	9 (24.3%)	3 (21.4%)	6 (26.1%)	0.757
No relevant thrombosis	2 (5.4%)	2 (14.3%)	0	0.065
Post-TEVAR CTA	n = 36	n = 13	n = 23	
Complete occlusion	27 (73.0%)	12 (92.3%)	15 (65.2%)	0.170
<50% residual perfusion	6 (16.2%)	0	6 (26.1%)	0.065
>50% residual perfusion	3 (8.1%)	1 (7.7%)	2 (8.7%)	0.920
No relevant thrombosis	0	0	0	
Endoleaks				
Type Ib	4 (10.8%)	1 (7.1%)	3 (13.0%)	0.564
Type II	3 (8.1%)	1 (7.1%)	2 (8.7%)	0.871
Type III	0	0	0	
dSINE	0	0	0	
CTA at Follow-up	n = 32	n = 10	n = 22	
Complete occlusion	29 (90.6%)	8 (80.0%)	21 (95.5%)	0.920
<50% residual perfusion	3 (9.4%)	2 (20.0%)	1 (4.5%)	0.175
>50% residual perfusion	0	0	0	
No relevant thrombosis	0	0	0	
Endoleak				
Type Ib	4 (10.8%)	2 (20.0%)	2 (9.1%)	0.429
Type II	1 (2.7%)	0	1 (4.5%)	0.329
Type III	1 (2.7%)	1 (10.0%)	0	0.347
dSINE	1 (2.7%)	0	1 (4.5%)	0.329

Values are n (%). CTA, computed tomography angiography; dSINE, distal stent-induced new entry; TEVAR, thoracic endovascular aortic repair.

## Data Availability

The data presented in this study are available on request from the corresponding author.

## Abbreviations

The following abbreviations are used in this manuscript:

CSF	Cerebrospinal fluid
CTA	Computed tomography angiography
DOAJ	Directory of open access journals
dSINE	Distal stent-induced new entry
DTA	Descending thoracic aorta
EC	Early completion
FET	Frozen elephant trunk
LC	Late completion
LSA	Left subclavian artery
MDPI	Multidisciplinary Digital Publishing Institute
TEVAR	Thoracic endovascular aortic repair
